# Retraction: Antioxidant Properties and Gastroprotective Effects of 2-(Ethylthio)Benzohydrazones on Ethanol-Induced Acute Gastric Mucosal Lesions in Rats

**DOI:** 10.1371/journal.pone.0294011

**Published:** 2023-11-10

**Authors:** The PLOS ONE Editors

Following the publication of this article [1], concerns were raised regarding reuse of results presented in Figs 4 and 7. Specifically,

- Fig 4c of this study [1] appears to partially overlap with Fig. 11c of [2], despite being used to represent different experimental conditions.
- Fig 7c of this study [1] appears to partially overlap with Figure 5c of [3, corrected in 4]*, despite being used to represent different experimental conditions.

The authors’ response did not resolve the editorial concerns and the original data underlying this study were not provided for editorial review. Given the nature of the issues, the *PLOS ONE* Editors are concerned about the reliability of data management and/or reporting for this study [1].

In light of the above concerns, the *PLOS ONE* Editors retract this article.

Some figure panels discussed above appear to report previously published material that are offered under a CC BY license, but the original article was not attributed in [1]. For these images, the * by the citation, above, marks the oldest publication of the image of which PLOS is aware.

All authors agreed with the retraction. MAA stands by the article’s findings.

## References

[1] Nazarbahjat N, Kadir FA, Ariffin A, Abdulla MA, Abdullah Z, Yehye WA (2016) Antioxidant Properties and Gastroprotective Effects of 2-(Ethylthio)Benzohydrazones on Ethanol-Induced Acute Gastric Mucosal Lesions in Rats. PLoS ONE 11(6): e0156022. doi: 10.1371/journal.pone.0156022 27272221 PMC4896442

[2] Salama SM, Gwaram NS, AlRashdi AS, Khalifa SAM, Abdulla MA, Ali HM, and El-Seedi HR (2016) A Zinc Morpholine Complex Prevents HCl/Ethanol-Induced Gastric Ulcers in a Rat Model. Scientific Reports, 6: 29646. doi: 10.1038/srep29646 27460157 PMC4962080

[3] Ismail IF, Golbabapour S, Hassandarvish P, Hajrezaie M, Majid NA, Kadir FA, et al. (2012) Gastroprotective Activity of *Polygonum chinense* Aqueous Leaf Extract on Ethanol-Induced Hemorrhagic Mucosal Lesions in Rats. Evidence-Based Complementary and Alternative Medicine, Volume 2012, Article ID 404012. doi: 10.1155/2012/404012 23365597 PMC3544547

[4] Ismail IF, Golbabapour S, Hassandarvish P, Hajrezaie M, Majid NA, Kadir FA, et al. (2018) Corrigendum to “Gastroprotective Activity of *Polygonum chinense* Aqueous Leaf Extract on Ethanol-Induced Hemorrhagic Mucosal Lesions in Rats”. Evidence-Based Complementary and Alternative Medicine, Volume 2018, Article ID 8961462. doi: 10.1155/2018/8961462 30647764 PMC6320006

